# Enhancing the electronic and optical properties of the metal/semiconductor NbS_2_/BSe nanoheterostructure towards advanced electronics

**DOI:** 10.1039/d3na01086d

**Published:** 2024-01-30

**Authors:** S. T. Nguyen, T. T. T. Huong, N. X. Ca, C. Q. Nguyen

**Affiliations:** a Faculty of Electrical Engineering, Hanoi University of Industry Ha Noi 100000 Vietnam nguyensontung@haui.edu.vn; b Institute of Science and Technology, TNU-University of Sciences Thai Nguyen Vietnam canx@tnus.edu.vn; c Department of Science and Technology, Ha Noi University of Industry Ha Noi 100000 Vietnam; d Institute of Research and Development, Duy Tan University Da Nang 550000 Vietnam nguyenquangcuong3@duytan.edu.vn; e Faculty of Natural Sciences, Duy Tan University Da Nang 550000 Vietnam

## Abstract

Metal–semiconductor (M–S) contacts play a vital role in advanced applications, serving as crucial components in ultracompact devices and exerting a significant impact on overall device performance. Here, in this work, we design a M–S nanoheterostructure between a metallic NbS_2_ monolayer and a semiconducting BSe monolayer using first-principles prediction. The stability of such an M–S nanoheterostructure is verified and its electronic and optical properties are also considered. Our results indicate that the NbS_2_/BSe nanoheterostructure is structurally, mechanically and thermally stable. The formation of the NbS_2_/BSe heterostructure leads to the generation of a Schottky contact with the Schottky barrier ranging from 0.36 to 0.51 eV, depending on the stacking configurations. In addition, the optical absorption coefficient of the NbS_2_/BSe heterostructure can reach up to 5 × 10^5^ cm^−1^ at a photon energy of about 5 eV, which is still greater than that in the constituent NbS_2_ and BSe monolayers. This finding suggests that the formation of the M–S NbS_2_/BSe heterostructure gives rise to an enhancement in the optical absorption of both NbS_2_ and BSe monolayers. Notably, the tunneling probability and the contact tunneling-specific resistivity at the interface of the NbS_2_/BSe heterostructure are low, indicating its applicability in emerging nanoelectronic devices, such as Schottky diodes and field-effect transistors. Our findings offer valuable insights for the practical utilization of electronic devices based on the NbS_2_/BSe heterostructure.

## Abstract 2

Metal–semiconductor (M–S) contacts play a vital role in advanced applications, serving as crucial components in ultracompact devices and exerting a significant impact on overall device performance.

## Graphical abstract

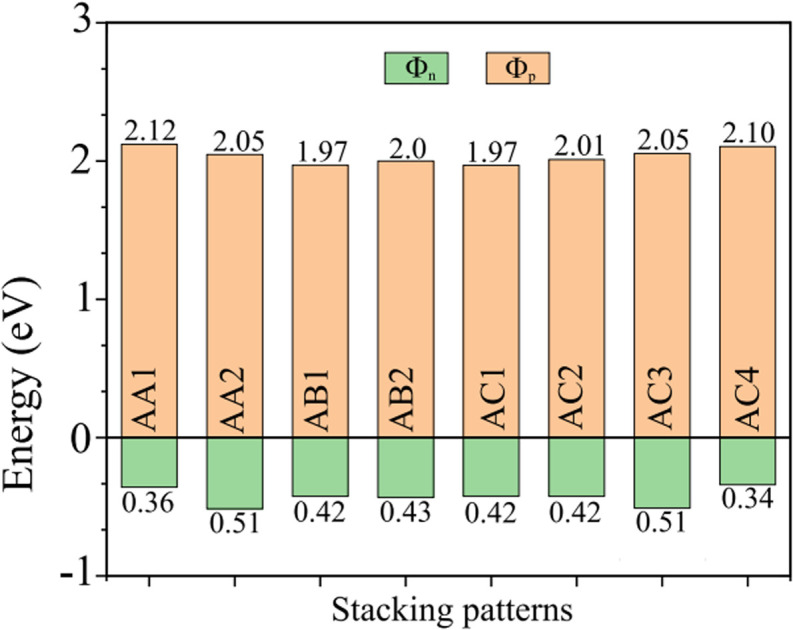

## 1 Introduction

Two-dimensional (2D) materials, including graphene,^1^ phosphorene^2^ and transition metal dichalcogenides,^3^ have received much attention from the research community owing to their exceptional properties and diverse potential applications. As the first successfully fabricated 2D material, graphene emerges as a promising candidate in various next-generation applications, including electronic,^4^ optoelectronic^5^ and spintronic^6^ applications. However, the absence of a desirable band gap in graphene is crucial, especially in the design of high-frequency applications, where it plays a significant role. Hence, many different strategies, such as doping,^7^ functionalization^8^ and strain^9^ have been proposed to open an intrinsic band gap in graphene. It's worth noting that these strategies may potentially result in a reduction in the carrier mobility of graphene.^10^ Therefore, seeking 2D materials with an appropriated band gap for high-speed applications remains a challenging endeavor.

Recently, the construction of a van der Waals (vdW) heterostructure by stacking two or more 2D materials has emerged as a common strategy to improve the properties and extend the application possibilities of 2D materials.^11,12^ VdW heterostructures can be synthesized in experiments by the chemical vapor deposition (CVD) method,^13,14^ one-step growth^15^ or two-step growth.^16^ Additionally, these heterostructures also can be predicted through first-principles calculations.^17,18^ Following the success of both experimental and theoretical endeavors, many different vdW heterostructures have been predicted and synthesized, such as graphene-based heterostructures,^19–23^ phosphorene-based heterostructures^24–26^ and MA_2_Z_4_-based heterostructures.^27–29^ The 2D vdW heterostructures can be categorized into metal/semiconductor (M–S) and semiconductor/semiconductor heterostructures (S–S), which depend on the characteristics of 2D materials. It's worth noting that the M–S heterostructure is one of the most crucial components in electronic devices. Hence, the characteristics of the M–S heterostructure significantly impact the performance of electronic devices. Therefore, there is growing attention on the search for M–S heterostructures towards next-generation electronic devices aiming for high performance.

Recently, many different M–S heterostructures have been proposed and investigated, involving the combination of both traditional 3D metals or novel 2D metals with 2D semiconductors, such as 3D and 2D metals/MSi_2_N_4_ (M = Mo, W)^30^ and 3D metals/MoS_2_.^31^ Notably, the traditional 3D metals/semiconductor often exhibit unmodulated Schottky barriers, leading to high contact resistance and lower carrier mobility, thus diminishing device performance. In contrast, the 2D metals/semiconductor heterostructures may feature lower Schottky barriers and they are also tunable under various conditions, including strain and electric fields.^32–36^ Hence, there is a growing focus on the discovery of novel 2D metals and semiconductors, which can be combined to form an M–S contact with enhanced performance.

Currently, a 2D metal NbS_2_ monolayer has received tremendous consideration as it can serve as a promising metallic electrode for novel M–S heterostructures.^37–40^ 2D metallic NbS_2_ was synthesized in recent experiments by different methods, such as chemical vapor deposition^41,42^ and mechanical/chemical exfoliation.^43^ The electronic and optical properties of the NbS_2_ monolayer have also been investigated through first-principles calculations^44^ along with its tunable properties *via* doping^45^ and intercalations.^46^ All these findings suggest that a metallic NbS_2_ monolayer can be considered as a promising material for future applications, including gas sensors^47,48^ and energy storage.^49^ Furthermore, a promising BSe semiconducting monolayer has been predicted to be mechanically and thermally stable at room temperature.^50^ It is evident that the BSe monolayer exhibits a semiconducting feature with an indirect band gap. Additionally, the BSe monolayer is considered as a promising semiconductor for forming contacts with various other 2D materials such as graphene,^51^ phosphorene,^52^ MoS_2_ (ref. 17) and so forth.^53,54^ However, to date, a combination between a BSe semiconductor and other 2D metals has not yet been extensively predicted and investigated. Therefore, in this work, we perform first-principles calculations to design a M–S heterostructure by combining 2D metal NbS_2_ and a BSe semiconductor. Our findings offer valuable insights for the practical utilization of electronic devices based on the NbS_2_/BSe heterostructure.

## 2 Computational model and methods

In this work, we performed first-principles calculations based on the density functional theory (DFT). We use the Quantum Espresso simulation package^55^ in the framework of the generalized gradient approximation (GGA).^56^ In addition, we employed the Perdew–Burke–Ernzerhof (PBE) functional^57^ to calculate the exchange and correlation energy with the projected augmented wave (PAW) pseudopotential.^58^ The vdW interactions that may exist in layered nanostructures can be described by using the Grimme correction DFT-D3 method.^59^ The Heyd–Scuseria–Ernzerhof (HSE) functional^60^ is also used to achieve more accurate band gaps of 2D semiconductors. A cut-off energy of 510 eV and a (9 × 9 × 1) *k*-point mesh are used in all our calculations. A vacuum thickness of 30 Å is included to prevent unnecessary interactions caused by the periodic boundary conditions. Dipole correction is also applied in all calculations.

## 3 Results and discussion

**Fig. 1:**
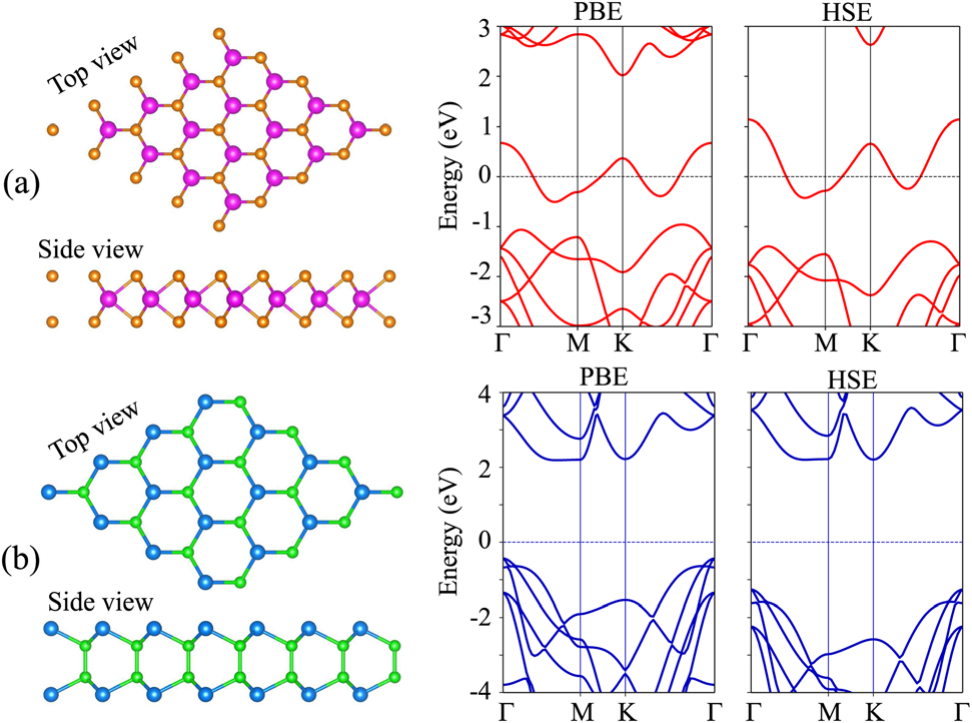
(From left to right) Atomic structure, PBE and HSE band structures of (a) NbS_2_ and (b) BSe monolayers. Orange and purple balls represent the S and Nb atoms, respectively. Dark green and green balls stand for the Se and B atoms, respectively.

We first examine the atomic and electronic structures of NbS_2_ and BSe monolayers, as illustrated in Fig. 1. Both the NbS_2_ and BSe monolayers show hexagonal atomic crystals. In the NbS_2_ monolayer, one Nb atom is sandwiched between two S atoms on both sides, whereas in the BSe monolayer, each B atom is bonded with one Se atom on one side. The calculated lattice parameters of the NbS_2_ and BSe monolayers are 3.31 and 3.23 Å, respectively. These results are in good agreement with previous reports.^50,61^ The NbS_2_ monolayer is characterized by its metallic properties, featuring a band that crosses the Fermi level. In contrast, the BSe monolayer exhibits a semiconducting behavior with an indirect band gap of 2.62/3.46 eV, obtained by using the PBE/HSE functional. These values are in good agreement with previous measurements.^50^ The valence band maximum (VBM) and conduction band minimum (CBM) of the BSe monolayer are located at the *Γ* point and *Γ*–*M* path, respectively. Interestingly, we find that both the PBE and HSE functionals predict the similar behavior of NbS_2_ and BSe monolayers. The main difference between PBE and HSE methods is in a shift of the VBM of the BSe monolayer. The VBM of the BSe monolayer in the PBE functional is positioned closer to the Fermi level than that in the HSE functional.

**Fig. 2:**
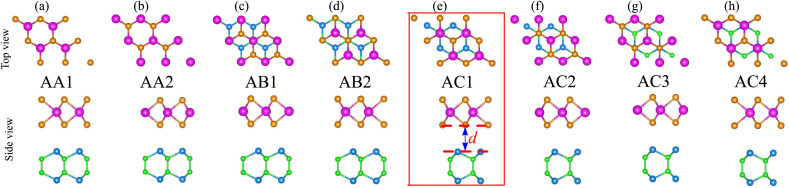
Atomic structures of the NbS_2_/BSe heterostructure for possible different stacking configurations of (a) AA1, (b) AA2, (c) AB1, (d) AB2, (e) AC1, (f) AC2, (g) AC3 and (h) AC4.

We now combine the NbS_2_ and BSe monolayers to generate the metal/semiconductor NbS_2_/BSe heterostructure by stacking NbS_2_ on top of the BSe monolayer. The possible stacking configurations of the NbS_2_/BSe heterostructure are depicted in Fig. 2. Due to a small difference in the lattice parameters between NbS_2_ and BSe monolayers, the NbS_2_/BSe heterostructure has a small lattice mismatch of only 1.2% for all stacking configurations. After the geometric optimization process, the interlayer spacing *d* between NbS_2_ and BSe layers in their heterostructure can be obtained, as listed in Table 1. It is evident that the interlayer spacing in the AC1 stacking configuration is *d* = 2.95 Å, which is the shortest among the eight stacking configurations. In addition, it should be noted that the interlayer spacing *d* in all stacking configurations is larger than the sum of covalent radii between S (0.99 Å) and Se (1.14 Å) atoms, confirming that there is no covalent bond between the two constituent NbS_2_ and BSe layers. Furthermore, to examine the interfacial stability of the NbS_2_/BSe heterostructure for all stacks, we calculate the binding energy as follows:1
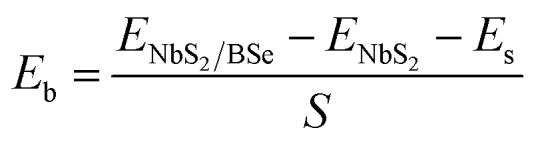
Here, *E*_NbS_2_/BSe_, *E*_NbS_2__ and *E*_BSe_ are the total energies of the NbS_2_/BSe heterostructure, and isolated NbS_2_ and BSe monolayers, respectively. *S* = 10.69 Å^2^ is the surface area of the heterostructure. The calculated *E*_b_ for all stacks of the NbS_2_/BSe heterostructure is listed in Table 1. The binding energy of the NbS_2_/BSe heterostructure is approximately averaged at about −20 meV Å^−2^. The negative binding energy suggests that the NbS_2_/BSe heterostructure is stable. Interestingly, this binding energy is comparable to that in graphite^62^ and other typical 2D van der Waals (vdW) heterostructures.^63,64^ This finding indicates that the NbS_2_/BSe heterostructure can be synthesized in future experiments by different strategies, including mechanical exfoliation^12^ and CVD.^65^ For instance, using the CVD method, Fu *et al.*^37^ successfully synthesized a metal/semiconductor NbS_2_/MoS_2_ heterostructure with high quality and a clean interface. Among these stacks, the AC2 stack has the lowest binding energy of −23.89 meV Å^−2^. The shortest interlayer spacing and the lowest binding energy in the AC1 stacking configuration of the NbS_2_/BSe heterostructure indicate that the AC1 stack is the most energetically feasible stacking configuration.

**Table 1:** Calculated interlayer spacing (*d*, Å), binding energy (*E*_b_, meV Å^−2^), and Schottky barrier heights (*Φ*_n_ and *Φ*_p_, eV) of the NbS_2_/BSe heterostructure

Stacks	*D*	*E*_b_	*Φ*_n_	*Φ*_p_	Contact types
AA1	3.11	−22.16	2.12	0.36	p-type ShC
AA2	3.58	−14.50	2.05	0.51	p-type ShC
AB1	2.95	−23.88	1.97	0.42	p-type ShC
AB2	3.01	−23.47	2.0	0.43	p-type ShC
AC1	2.95	−23.89	1.97	0.42	p-type ShC
AC2	3.01	−23.47	2.01	0.42	p-type ShC
AC3	3.58	−14.67	2.05	0.51	p-type ShC
AC4	3.04	−23.01	2.10	0.34	p-type ShC

**Fig. 3:**
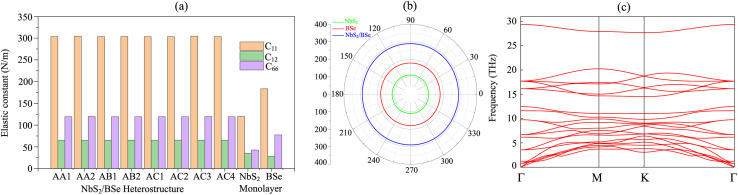
(a) Calculated independent elastic constants (*C*_*ij*_) of the NbS_2_/BSe heterostructure for different stacking configurations and (b) angle-dependent Young's modulus and (c) phonon dispersion curves of the NbS_2_/BSe heterostructure for the most energetically favorable stacking configuration.

Furthermore, to check the mechanical stability of the NbS_2_/BSe heterostructure, we consider its independent elastic constants (*C*_*ij*_). The *C*_*ij*_ of the perfect NbS_2_ and BSe monolayers are also calculated for comparison, as illustrated in Fig. 3. The calculated elastic constants *C*_11_, *C*_12_ and *C*_66_ of monolayers NbS_2_ and BSe are 183.31, 28.40, 77.45 and 120.10, 34.91 and 42.59 N m^−1^, respectively. According to the Born–Huang stability criteria,^66^*i.e. C*_11_ > *C*_12_, *C*_66_ > 0 and *C*_11_^2^ – *C*_12_^2^ > 0, both the NbS_2_ and BSe monolayers are mechanically stable. Interestingly, the formation of the NbS_2_/BSe heterostructure leads to an increase in the elastic constants *C*_*ij*_, as shown in Fig. 3(a). The calculated *C*_11_, *C*_12_ and *C*_66_ of the NbS_2_/BSe heterostructure are about 300, 65 and 130 N m^−1^, respectively. These values of the elastic constants are still larger than those of the NbS_2_ and BSe monolayers. In addition, the elastic constants of the NbS_2_/BSe heterostructure meet the Born–Huang stability criteria. This finding suggests that the NbS_2_/BSe heterostructure exhibits outstanding mechanical stability. Furthermore, the angle-dependent Young's modulus of NbS_2_ and BSe monolayers and their combined NbS_2_/BSe heterostructure for the most energetically favorable stacking configuration are depicted in Fig. 3(b). We find that the Young's modulus of the NbS_2_/BSe heterostructure is calculated to be 289.74 N m^−1^, which is greater than that of the NbS_2_ (109.95 N m^−1^) and BSe (178.90 N m^−1^) monolayers. The phonon dispersion curves of the NbS_2_/BSe heterostructure are depicted in Fig. 3(c). We observe that there are no imaginary frequencies in the phonon spectrum of the NbS_2_/BSe heterostructure, indicating that such a heterostructure is dynamically stable. All these findings indicate that the NbS_2_/BSe heterostructure can be considered as a promising candidate for the design of electronic devices.

**Fig. 4:**
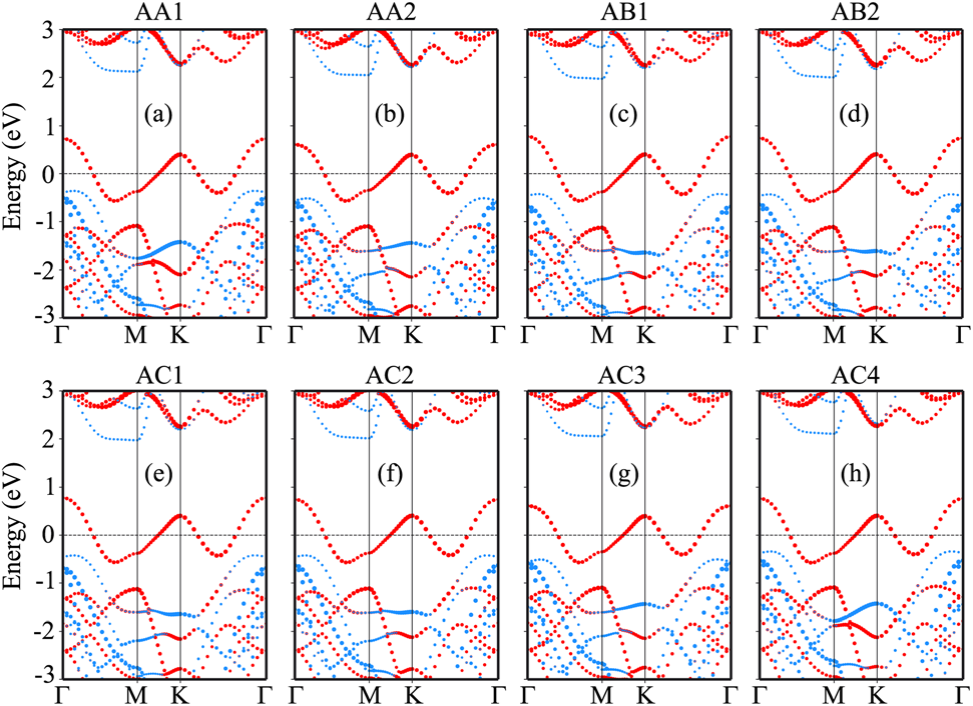
Projected band structures of the NbS_2_/BSe heterostructure for different stacks of (a) AA1, (b) AA2, (c) AB1, (d) AB2, (e) AC1, (f) AC2, (g) AC3 and (h) AC4. Red and blue bubbles represent the contributions of the NbS_2_ and BSe layers, respectively.

The projected band structures of the NbS_2_/BSe heterostructure for all stacks are depicted in Fig. 4, in which red and blue bubbles show the contributions of the NbS_2_ and BSe layers, respectively. It is obvious that the band structures of the NbS_2_/BSe heterostructure appear to be a combination of the band structures of the constituent monolayers. The reason for such a combination is weak interactions between the NbS_2_ and BSe layers. These weak interactions play a pivotal role in stabilizing the heterostructure, making it readily obtainable in experiments. Furthermore, the combination between the metallic NbS_2_ monolayer and semiconducting BSe monolayer might give rise to the formation of either a Schottky or ohmic contact, depending on the position of the band edges of the BSe semiconductor in relation to the Fermi level of the metallic NbS_2_ layer. As depicted in Fig. 4, the Fermi level of the metallic NbS_2_ monolayer lies between the band edges of the semiconductor BSe monolayer. This arrangement leads to the formation of a Schottky contact. In a Schottky contact, Schottky barriers exist, and these barriers can be determined as follows:

2 *Φ*_n_ = *E*_CBM_ − *E*_F_

and

3 *Φ*_p_ = *E*_F_ − *E*_VBM_

**Fig. 5:**
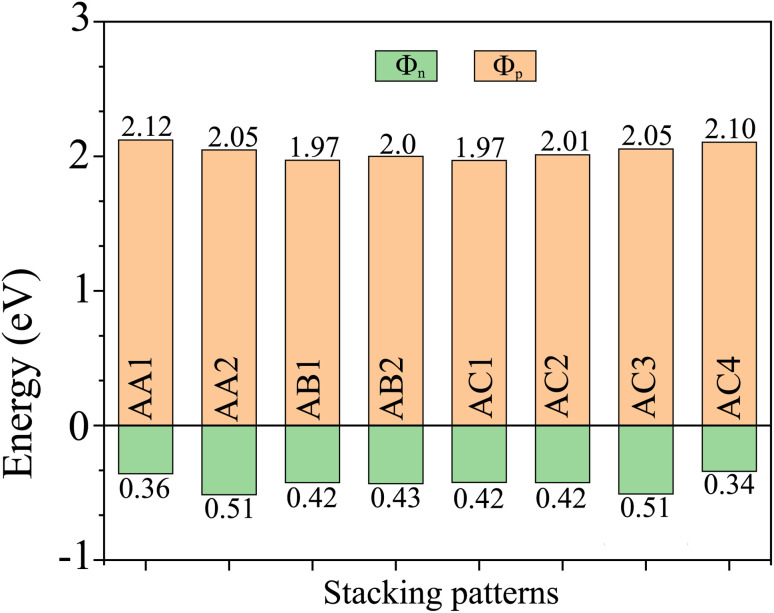
The Schottky barriers of the NbS_2_/BSe heterostructure for different stacking patterns.

Here, *E*_CBM_ and *E*_VBM_ are the band edge energies of the CBM and VBM of the BSe semiconductor, respectively. *E*_F_ is the Fermi level of the heterostructure. The obtained Schottky barriers *Φ*_n_ and *Φ*_p_ of the heterostructure are listed in Table 1 and Fig. 5. It is evident that the Schottky barrier *Φ*_p_ of the heterostructure is always narrower than the Schottky barrier *Φ*_n_, indicating that the NbS_2_/BSe heterostructure tends to exhibit p-type ShC. The p-type Schottky barrier in the NbS_2_/BSe heterostructure ranges from 0.34 to 0.51 eV. The AC4 stacking pattern exhibits the narrowest Schottky barrier *Φ*_p_ of 0.34 eV, while the AA2 and AC3 stacking patterns exhibit the highest Schottky barrier *Φ*_p_ of 0.51 eV. As previously discussed, the AC1 stacking pattern of the NbS_2_/BSe heterostructure is the most favorable among the stacking configurations. Hence, we will focus on this stacking configuration in our subsequent calculations.

**Fig. 6:**
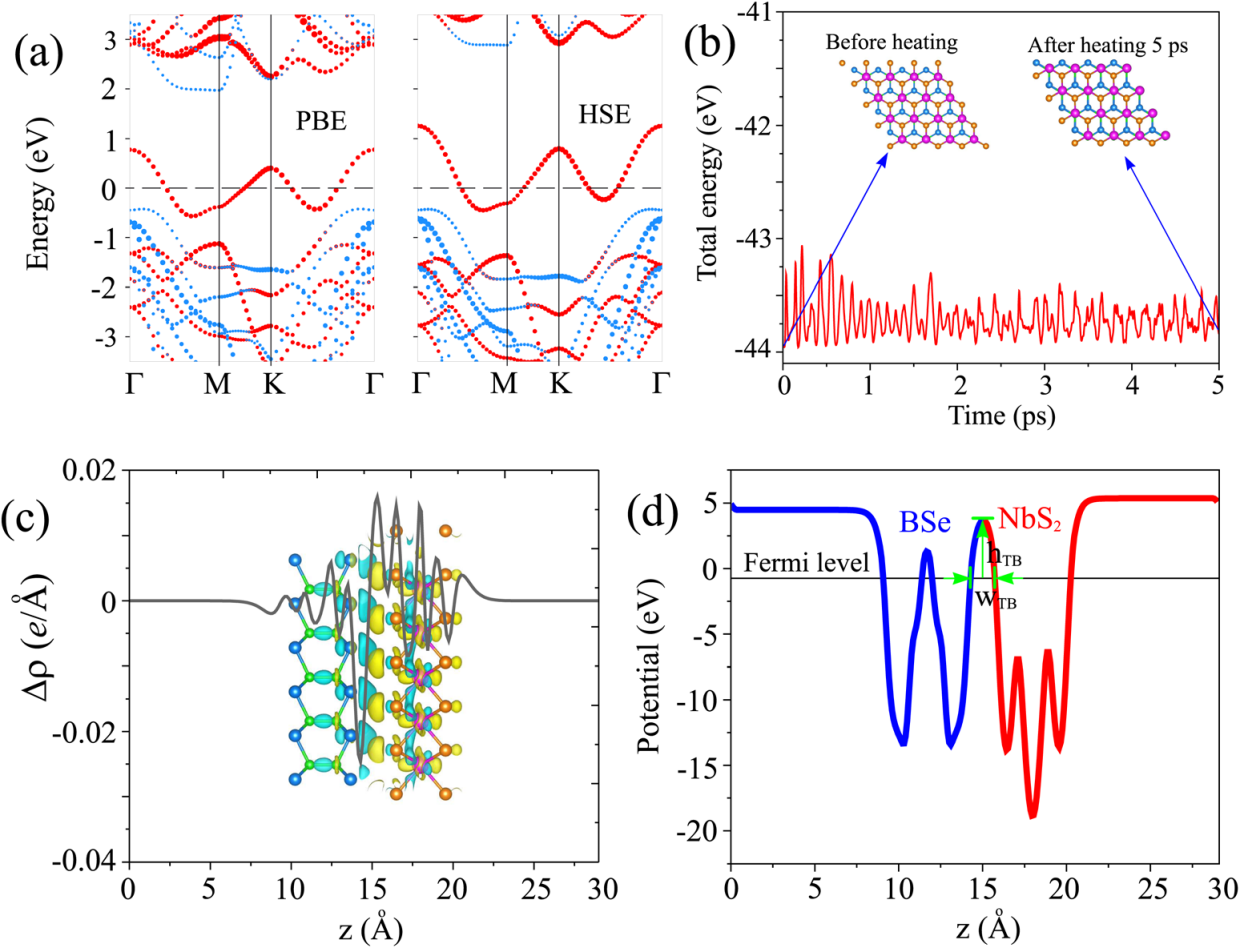
(a) Projected band structures, (b) AIMD simulation, (c) charge density difference and (d) electrostatic potential of the AC1 stacking pattern of the NbS_2_/BSe heterostructure. Red and blue lines represent the contribution of metallic NbS_2_ and semiconducting BSe monolayers, respectively. The inset of (c) shows the atomic structure of the heterostructure before and after heating for 5 ps. Yellow and cyan regions in (c) indicate the charge accumulation and depletion, respectively.

The projected band structures of the NbS_2_/BSe heterostructure for the most energetically favorable stacking patterns using PBE and HSE methods are illustrated in Fig. 6(a). The calculated Schottky barriers *Φ*_n_ and *Φ*_p_ of the NbS_2_/BSe heterostructure are 1.97/2.88 and 0.42/0.43 eV, obtained using the PBE/HSE functional. It is clear that both the PBE and HSE functionals predict the similar behavior of the NbS_2_/BSe heterostructure. The CBM calculated using the HSE functional of the NbS_2_/BSe heterostructure is positioned further away from the Fermi level than that calculated by using the PBE functional. Interestingly, it's worth noting that both the PBE and HSE functional predictions indicate a p-type Schottky contact with the same Schottky barrier *Φ*_p_. This suggests that the PBE functional can be effectively used in the subsequent calculations without compromising the accuracy of the results. Furthermore, in order to verify the thermal stability of the NbS_2_/BSe heterostructure, we perform *ab initio* molecular dynamics (AIMD) simulation. The AIMD simulation of the NbS_2_/BSe heterostructure is displayed in Fig. 6(b). It is evident that the fluctuation in the total energy of the NbS_2_/BSe heterostructure as a function of time steps is small. In addition, the atomic structure of the NbS_2_/BSe heterostructure after heating for 5 ps is preserved without any distortion. All these findings confirm that the NbS_2_/BSe heterostructure is thermally stable. The stability indicates that the NbS_2_/BSe heterostructure could be synthesized and used in recent nanoelectronic and optoelectronic devices, such as Schottky diodes, field-effect transistors and photodetectors.

Furthermore, the charge transfers between the NbS_2_ and BSe layers in their heterostructure are also explored by calculating the charge density difference (CDD) as follows:

4 Δ*ρ* = *ρ*_NbS_2_/BSe_ − *ρ*_NbS_2__ − *ρ*_BSe_

Here, the charge densities of the NbS_2_/BSe heterostructure and isolated NbS_2_ and BSe monolayers are denoted as *ρ*_NbS_2_/BSe_, *ρ*_NbS_2__ and *ρ*_BSe_, respectively. The CDD of the NbS_2_/BSe heterostructure is depicted in Fig. 6(c). One can find that the charges are accumulated mainly around the NbS_2_ layer and depleted mainly around the BSe layer. This finding implies that the electrons are transferred from the NbS_2_ to the BSe layer in their corresponding NbS_2_/BSe heterostructure. Based on Bader charge analysis, there is a small amount of charge transfer of only 0.02 electrons that flow from the NbS_2_ to the BSe layer.

The electrostatic potential of the NbS_2_/BSe heterostructure is depicted in Fig. 6(d). One can observe that the NbS_2_ layer has a deeper potential that the BSe layer in their heterostructure. The charge transfer between metallic NbS_2_ and BSe layers at the interface of the NbS_2_/BSe heterostructure gives rise to the formation of an interfacial dipole, which can be obtained using Δ*V* = *W*_NbS_2__ − *W*_NbS_2_/BSe_, where the work functions of the metallic NbS_2_ layer and the NbS_2_/BSe heterostructure are denoted as the *W*_NbS_2__ and *W*_NbS_2_/BSe_, respectively. The interfacial dipole at the interface of the NbS_2_/BSe heterostructure is obtained to be 0.02 eV. The formation of the interfacial dipole at the interface can change the Schottky barriers. However, our results show that both the amount of charge transfer and interface dipole are small; hence, the change in the Schottky barriers at the interface of the NbS_2_/BSe heterostructure can be considered negligible. Furthermore, both the charge transfer and interfacial dipole in the NbS_2_/BSe heterostructure may give rise to the formation of a built-in electric field at the interface. The built-in electric field can be calculated as follows:^67^5
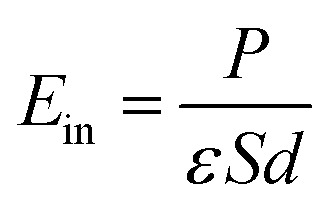
where *P* stands for the interface dipole. *ε*, *S* and *d* are the dielectric constant, surface area and interlayer distance of the NbS_2_/BSe heterostructure, respectively. We can observe that the built-in electric field is directly proportional to the interface dipole. As we have discussed above, the amount of charge transfer and interface dipole of the NbS_2_/BSe heterostructure are small; hence, the built-in electric field across the interface of the heterostructure is also small and can be considered negligible.

Furthermore, in order to evaluate the efficiency of electron injection through the contact of the NbS_2_/MoSSe heterostructure, we add the calculations on the tunneling probability 
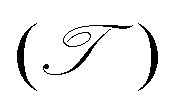
 and the tunneling-specific resistivity (*ρ*_t_) as follows:6
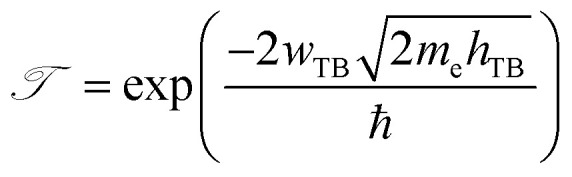
and7
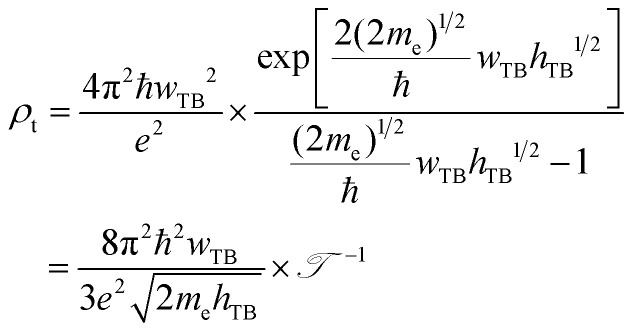
Here, ℏ is the reduced Planck's constant. *e* and *m*_e_ are the electron magnitude and mass of a free electron, respectively. *h*_TB_ and *w*_TB_ are the tunneling barrier height and width, respectively, which can be obtained directly from the electrostatic potential of the NbS_2_/BSe heterostructure. The calculated *h*_TB_ and *w*_TB_ of the NbS_2_/BSe heterostructure for the most energetically favorable stacking configuration are 3.68 eV and 1.35 Å, respectively. Hence, the tunneling probability and contact tunneling-specific resistivity at the interface of the NbS_2_/BSe heterostructure are about 7% and 2.09 × 10^−10^ Ω cm^2^, respectively. We also observe that the value of the tunneling-specific resistivity at the interface of the NbS_2_/MoSSe heterostructure exhibits a magnitude similar to that in other metal/semiconductor contacts, such as Bi/MoS_2_,^68^ semimetals/TMDs,^69^ metal/MSi_2_N_4_ (M = Mo, W)^29^ and 2D (3D) metals/GeSe.^70^ This finding also suggests that the NbS_2_/BSe heterostructure could serve as an efficient contact for electronic devices.

**Fig. 7:**
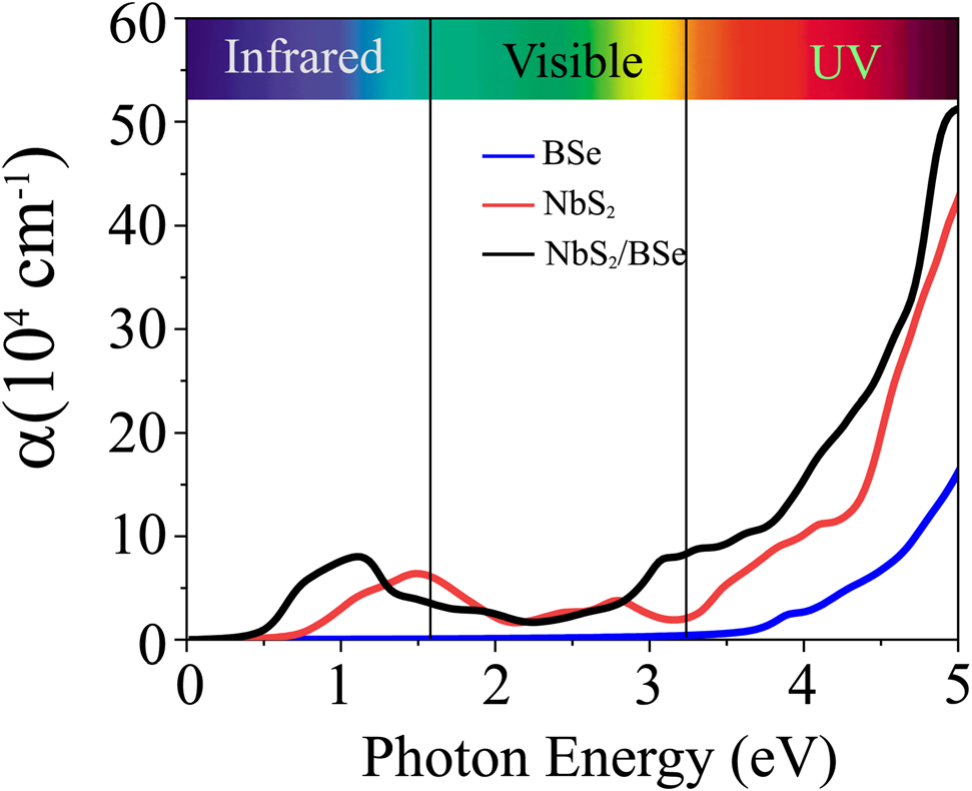
Calculated optical absorption as a function of the photon energy of the NbS_2_/BSe heterostructure and the constituent BSe and NbS_2_ monolayers.

Furthermore, we calculate the optical absorption of the NbS_2_/BSe heterostructure as well as that of the constituent NbS_2_ and BSe monolayers for comparison. The optical absorption can be obtained as follows:8
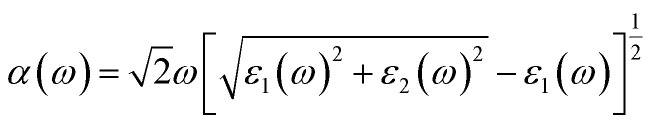
Here, the real and imaginary parts of dielectric functions are denoted ass *ε*_1_ and *ε*_2_, respectively. The optical absorption of the NbS_2_/BSe heterostructure and the constituent NbS_2_ and BSe monolayers are depicted in Fig. 7. We can find that the optical absorption coefficient of the NbS_2_/BSe heterostructure can reach up to 5 × 10^5^ cm^−1^ at a photon energy of about 5 eV, which is still greater than that in the constituent NbS_2_ and BSe monolayers. Hence, the formation of the M–S NbS_2_/BSe heterostructure gives rise to an enhancement in the optical absorption of both NbS_2_ and BSe monolayers. The enhancement in the optical absorption of the M–S NbS_2_/BSe heterostructure indicates that it can be considered as a promising candidate for the design of optoelectronic devices.

## 4 Conclusions

In summary, we have designed a metal/semiconductor heterostructure between two different materials 2D NbS_2_ metal and 2D BSe semiconductor using first-principles prediction. The combination between NbS_2_ and BSe monolayers gives rise to the formation of a metal/semiconductor NbS_2_/BSe heterostructure with different stacking configurations. All these stacking configurations of the NbS_2_/BSe heterostructure are considered to be structurally, mechanically and thermally stable, suggesting their potential as components in electronic devices. The formation of the NbS_2_/BSe heterostructure leads to the generation of a Schottky contact with the Schottky barrier ranging from 0.36 to 0.51 eV, depending on the stacking configurations. In addition, the optical absorption coefficient of the NbS_2_/BSe heterostructure can reach up to 5 × 10^5^ cm^−1^ at a photon energy of about 5 eV, which is still greater than that in the constituent NbS_2_ and BSe monolayers. This finding suggests that the formation of the M–S NbS_2_/BSe heterostructure gives rise to an enhancement in the optical absorption of both NbS_2_ and BSe monolayers. Notably, the tunneling probability and the contact tunneling-specific resistivity at the interface of the NbS_2_/BSe heterostructure are low, indicating its applicability in emerging nanoelectronic and optoelectronic devices, such as Schottky diodes, field-effect transistors and photodetectors. Our findings offer valuable insights for the practical utilization of electronic and optoelectronic devices based on the NbS_2_/BSe heterostructure.

## Conflicts of interest

There are no conflicts to declare.

## Supplementary Material
